# Development of New Natural Lipid-Based Nanoparticles Loaded with Aluminum-Phthalocyanine for Photodynamic Therapy against Melanoma

**DOI:** 10.3390/nano12203547

**Published:** 2022-10-11

**Authors:** Victor Carlos Mello, Victor Hugo Sousa Araújo, Karen Letycia Rodrigues de Paiva, Marina Mesquita Simões, Dafne Caroline Marques, Nelice Roberta da Silva Costa, Isadora Florêncio de Souza, Patricia Bento da Silva, Igor Santos, Raquel Almeida, Kelly Grace Magalhães, Sebastião William da Silva, Alexandre Silva Santos, Fabiane Veiga-Souza, Paulo Eduardo Narcizo Souza, Marina Arantes Raddichi, João Paulo Figueiró Longo, Jennifer Thayanne Cavalcante de Araújo, Luis Alexandre Muehlmann, Marlus Chorilli, Sônia Nair Báo

**Affiliations:** 1 Postgraduate Program in Animal Biology, Department of Genetics and Morphology, Institute of Biological Sciences, University of Brasília, Brasília 70910-900, DF, Brazil; 2 Laboratory of Microscopy and Microanalysis, Department of Cell Biology, Institute of Biological Sciences, University of Brasília, Brasília 70910-900, DF, Brazil; 3 COOIL Institute, Brasília 72622-401, DF, Brazil; 4 School of Pharmaceutical Sciences, São Paulo State University, Araraquara 14800-903, SP, Brazil; 5 Laboratory of Nanobiotechnology, Department of Genetics and Morphology, Institute of Biological Sciences, University of Brasilia, Brasília 70910-900, DF, Brazil; 6 Laboratory of Immunology and Inflammation, Department of Cell Biology, University of Brasilia, Brasília 70910-900, DF, Brazil; 7 Optical Spectroscopy Laboratory, Institute of Physics, University of Brasilia, Brasília 70910-900, DF, Brazil; 8 Laboratory of Protein Chemistry and Biochemistry, Department of Cell Biology, Institute of Biology, University of Brasília, Brasília 70910-900, DF, Brazil; 9 Faculty of Ceilandia, University of Brasilia, Brasília 70910-900, DF, Brazil; 10 Laboratory of Electron Paramagnetic Resonance, Institute of Physics, University of Brasília, Brasília 70910-900, DF, Brazil

**Keywords:** third-generation photosensitizers, drug delivery systems, cancer, photodynamic therapy, Amazon butters

## Abstract

Photodynamic therapy (PDT) mediated by photosensitizers loaded in nanostructures as solid lipid nanoparticles has been pinpointed as an effective and safe treatment against different skin cancers. Amazon butters have an interesting lipid composition when it comes to forming solid lipid nanoparticles (SLN). In the present report, a new third-generation photosensitizing system consisting of aluminum-phthalocyanine associated with Amazon butter-based solid lipid nanoparticles (SLN-AlPc) is described. The SLN was developed using murumuru butter, and a monodisperse population of nanodroplets with a hydrodynamic diameter of approximately 40 nm was obtained. The study of the permeation of these AlPc did not permeate the analyzed skin, but when incorporated into the system, SLN-AlPc allowed permeation of almost 100% with 8 h of contact. It must be emphasized that SLN-AlPc was efficient for carrying aluminum-phthalocyanine photosensitizers and exhibited no toxicity in the dark. Photoactivated SLN-AlPc exhibited a 50% cytotoxicity concentration (IC_50_) of 19.62 nM when applied to B16-F10 monolayers, and the type of death caused by the treatment was apoptosis. The exposed phospholipid phosphatidylserine was identified, and the treatment triggered a high expression of Caspase 3. A stable Amazon butter-based SLN-AlPc formulation was developed, which exhibits strong in vitro photodynamic activity on melanoma cells.

## 1. Introduction

Although it accounts for only 1% of all malignant skin tumors, melanoma represents the most aggressive and mortal form of skin cancer [1]. An important aspect of this disease is its frequent resistance to treatments. Depending on tumor characteristics such as location, stage, and genetic profile, therapeutic options may be surgical resection, chemotherapy, radiotherapy, immunotherapy, or photodynamic therapy (PDT) [2].

PDT involves three essential components: photosensitizer (PS), light, and oxygen [3,4]. Its action stems from the excitation of the PS by light at a specific wavelength [5]. The excited PS then forms reactive oxygen species (ROS) after interacting with molecular oxygen, triggering cytotoxic effects and leading to cell death by oxidative stress and the destruction of tumor microvasculature and activation of antitumor immunity [6]. Moreover, PDT can be used in combination with other therapeutic approaches such as: surgical removal, such as surgery, chemotherapy, radiotherapy, or immunotherapy [7].

The use of classical PDT against melanoma, however, has been limited by some issues. For example, melanoma can be resistant to PDT, mainly due to the presence of melanin pigment and melanosomes. For first-generation PS, which are activated by light sources with shorter wavelengths, these melanocyte pigments can be an obstacle for photon penetration in tissue and cell structures. Moreover, melanin is one of the stronger antioxidant molecules that we have in our body. Thus, as PDT is an oxidative therapy, the presence of these oxidative protective compounds makes the treatments even more challenging. The generation of ROS is the main process in PDT, which can lead to the destruction of tumor cells and further induce the immune response [8].

Therefore, several efforts were made to develop PS that are activated in longer wavelengths. Among them, the phthalocyanines are cited as an important representative of this group. For instance, phthalocyanine aluminum chloride (AlPc) absorbs light between 660 and 770 nm, has a high quantum fluorescence yield for the generation of singlet oxygen, and exhibits rapid and prolonged accumulation in cancer cells, as well as a reduced half-life time [9,10]. However, its low solubility in water is a substantial limitation for clinical applications, which impairs the administration and efficacy of the drug.

Moreover, despite these evolutions, some relevant drawbacks are related to second-generation PS. Their low aqueous solubility is probably one of the most relevant, which can promote challenges to bioviability and also some limitations in photodynamic activation due to PS aggregation. To overcome this, the PDT field proposed the association of second-generation PS with biocompatible PS nanocarriers, which are defined as third-generation PS, such as the PS that was developed in this report.

As research advances around the world, the understanding of molecular changes and mechanisms of melanoma develop, and new therapeutic approaches emerge, positively altering the treatment scenario of the disease [11]. Nanotechnology aims to improve adjuvant oncological approaches for melanoma, including PDT, by creating safe and effective drug delivery systems, for example, drugs with low aqueous solubility, such as AlPc, and may also increase selectivity in tumor cells. In the present study, Amazon butter-based solid lipid nanoparticles (SLNs) loaded with AlPc (SLN-AlPc) were used as a new third-generation PS system. SLNs are colloidal systems on a nanometer scale, consisting of solid lipids at room temperature, dispersed in an aqueous medium, and stabilized by a surfactant. They have high biocompatibility, high colloidal stability, and high capacity to carry lipophilic molecules such as AlPc in their oily center, in addition to having wide topical application [12]. Butters obtained from oilseeds have stood out as promising products for the development of SLNs due to the complexity of the lipid species that compose them, favoring more effective incorporation of drugs [13]. The main goal was to develop a third-generation photosensitizer based on aluminum-phthalocyanine (SLN-AlPc) loaded into SLNs, and to analyze their potential for photodynamic therapy in a melanoma (B16-F10) murine model. Moreover, the mechanisms of death, oxidative stress and activation of the immune system after the proposed treatment were evaluated. This formulation was effective as a photosensitizing system for PDT against B16-F10 cells in vitro [14].

## 2. Materials and Methods

### 2.1. Pre-Formulation Studies

The butters of murumuru (*Astrocaryum murumuru*), babassu (*Attalea speciosa*), bacuri (*Plantonia insignis*), and ucuuba (*Virola surinamensis*) were heated to 70 °C, and the crystalline events obtained after cooling were analyzed by polarized light microscopy (Leica DM2700 M, Leica Microsystems GmbH, Wetzlar, Germany) using 40× magnification.

The tested proportions were a 1:1 ratio of surfactant (Brij™ O10) to butter, corresponding to 5% (*w*/*v*) of the formulation, and the final concentration of AlPc was 20 μM and 40 μM. The samples were developed by heating the organic phase—composed of butter, surfactant, and AlPc—and water at 75 °C, separately. After complete melting of the butters under magnetic agitation of 500 rpm, the aqueous phase was transferred under organic, and agitation was maintained constant for 5 min. After this period, the system was subjected to magnetic agitation of 500 rpm, without heating, for 5 min until room temperature and formation of the SLN were reached.

### 2.2. Box–Behnken

Box–Behnken (BB) is widely used as a statistical tool for formulation optimization with a reduced number of experiments, and this model is also able to quantify the relationship between independent and dependent variables [15]. In this study, Box–Behnken was used to evaluate the impact of the variation between the proportion of murumuru butter and surfactant (Brij™ O10); the concentration of AlPc; and the agitation temperature in the parameters of HD, PdI, and ZP. The formulation development was based on the previously mentioned method. The variables’ coded values and actual values are described in Table 1.

Box–Behnken was designed by Minitab^®^ v. 19.1, State College, PA, USA, generating a total of 15 experiments, containing 3 central points and 3 levels (Table 2). The quadratic equation generated by the model is described below with Equation (1).

Y = b0 + b1A + b2B + b3C + b4A2 + b5B2 + b6C2 + b7AB + b8AC + b9BC(1)

where Y is the response obtained by measurement of the combination of the dependent variables; b0 to b9 are the regression coefficients of the variables and their interaction terms calculated from the experimental values. A, B, and C are the codes for independent variables, (butter and surfactant proportion, AlPc concentration, and temperature).

### 2.3. Evaluation of Encapsulation Efficiency (EE%)

The encapsulation efficiency was evaluated by the indirect method based on the studies by Araujo et al. (2020) [16], with modifications. The samples were centrifuged at 14,000 rpm for 30 min, and the supernatant that formed containing the nanostructures was collected. Then, the supernatant was diluted in methanol, which promoted the disruption of the nanosystem and the release of AlPc maintained inside. Subsequently, the formed solution was filtered through a 0.22 μm PTFE filter. The quantification was performed by HPLC, mobile phase 35:75 acidified water 2% (acetic acid) and anhydrous methanol (JTBaker®, Araraquara, Brazil), chromatographic column Zorbax SB-C8, the flow of 1 mL/min^−1^, column temperature of 30 °C, and detection at 670 nm using PDA module. The encapsulation efficiency was calculated according to Equation (2).

%EE = (obtained concentration)/(theorical concentration) × 100 (2)

### 2.4. Differential Scanning Calorimetry

DSC analysis was performed using a TG-DSC1 STARe system (Mettler Toledo, Columbus, OH, USA) in a nitrogen atmosphere. A heating rate of 10 °C/min^−1^ was adopted, with samples weighing around 10 mg and analyzed in alumina crucibles. For this technique, a temperature range of 25 °C to 400 °C was used. The evaluation of enthalpy variation (ΔH) and melting point was performed for the samples of murumuru butter, AlPc, SLN (drug-free nanosystem), and AlPc (nanosystem with 20 μM of AlPc).

### 2.5. Evaluation of Colloidal Properties

The mean particle size and polydispersity index (PDI) of SLNs were evaluated at 25 °C by photon correlation spectroscopy and electrophoretic laser Doppler velocimetry (ZetaSizer Nano ZS^®^, Malvern Instruments, Malvern, UK) with the angle of 90°. Before measurement, samples were diluted with distilled water (1:10, *v*:*v*).

### 2.6. Analytical Methodology

AlPc quantification was performed by high-performance liquid chromatography (HPLC) using the Waters™, e2695 instrument (Milford, MA, USA). The method developed was based on the one previously described by Muehlmann et al. (2015) [17]. The chromatographic condition used consisted of mobile phase 35:75 acidified water 2% (acetic acid) and anhydrous methanol (JTBaker^®^, Brazil), chromatographic column Zorbax SB-C8, the flow of 1 mL/min^−1^, column temperature of 30 °C, and detection at 670 nm using a PDA module. Linearity was measured by developing three analytical curves composed of nine different concentrations of AlPc (0.391, 0.781, 1.56, 3, 6.25, 12.5, 25, 50, and 100 μM).

The regression statistics were performed (correlation and determination coefficient), the curve equation was obtained by the least-squares method, and the significance of the angular and linear coefficients was evaluated.

### 2.7. In Vitro Skin Permeation

The assay was performed according to the methodology described by Vicenzo et al. (2022) [18] using Hanson Research microette Plus 60-301-106 permeator with 6 Franz cells. For this, pig ear skins (Frigodeliss, São Paulo, Brazil) were prepared using a TCM^®^ 300 dermatometer (Nouvag, Goldach, Switzerland) and hydrated with the receptor solution (phosphate buffer pH 7.4 with 5% Tween^®^ 20). AlPc in solution (absolute ethanol) and encapsulated in SLN (SLN-AlPc) were analyzed, according to the pre-established sink condition. The times established for sample collection were 0.50, 1, 2, 4, 6, 8, 10, 12, and 24 h. The samples were quantified in a spectrofluorimeter (Shimadzu RF-6000, Nakagyo-ku, Kyoto, Japan), using a calibration curve with a concentration range from 0.0005 to 5.0 μM.

### 2.8. Morphology of SLNs

The determination of SLN morphology was performed by transmission electron microscopy (TEM) using the equipment JEOL 1011 (Tokyo, Japan), which was operated at an accelerating voltage of 100 kV. The suspensions were diluted 1:10 (*v*:*v*) with Milli-Q^®^ water and deposited directly into the carbon-coated grids used for observation of the samples using osmium tetroxide. The microscope was operated in bright field mode with magnification above 10,000 times for morphological analysis.

### 2.9. Fourier-Transform InfraRed-FTIR

Fourier-transform infrared spectroscopy (Bruker Optik, Vertex-70, Ettlingen-Germany) was used to assess the inter-reactions between functional group level of the AlPc and the carrier system components. The technique was performed with attenuated total reflectance (ATR), with a spectral resolution of 4 cm^−1^, and 48 scans in the range from 400 to 4500 cm^−1^. The isolated components, solution of AlPc in alcohol, SLN formulation, physical mixture of AlPc with SLN formation (AlPc + SLN) and SLN-AlPc formulations were evaluated.

### 2.10. Raman Spectroscopy

Complementary to the FTIR-ATR, Raman spectroscopy was performed to evaluate the possible interactions between nanoparticle components. The analysis was performed with a resolution of 0.7 cm^−1^ in the range from 150 to 1800 cm^−1^ by the LabRam HR Evolution, Horiba. The samples were excited by the 532 nm line of a diode laser with power of 10 mW. The isolated components, solution of AlPc in alcohol, SLN formulation, physical mixture of AlPc with SLN formation (AlPc + SLN) and SLN-AlPc formulations were evaluated.

### 2.11. Surface-Enhanced Raman Spectroscopy-SERS

Silver films used as a substrate for SERS measurements were prepared by the electrolytic method using a solution of AgNO_3_ (0.1 mg/mL) as an electrolyte. Two silver rods (purity ≥ 99.9%) were immersed into the electrolyte as anode and cathode. A direct current was applied to the rods for 1 h, producing a surface nanosilver film. The silver rods were removed from the electrolyte and dried with nitrogen flux. The analysis was performed with a resolution of 0.7 cm^−1^ in the range from 150 to 1800 cm^−1^ by the LabRam HR Evolution, Horiba, Kyoto, Japan. The samples were excited by the 532 nm line of a diode laser with power of 10 mW. The isolated components, solution of AlPc in alcohol, SLN formulation, physical mixture of AlPc with SLN formation (AlPc + SLN), and SLN-AlPc formulations were evaluated.

### 2.12. In Vitro Experimental Design

**Figure 1:**
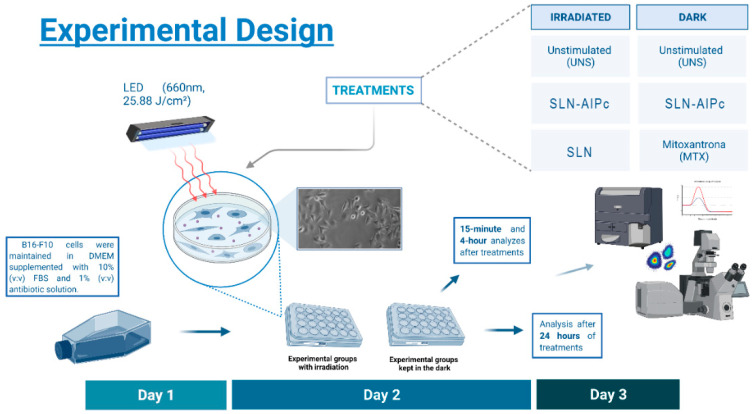
Experimental design for the in vitro analyses. Day 1: B16-F10 cells were cultivated for 24 h. Day 2: B16-F10 cells were washed in PBS and then treated with PDT. In the SLN-AlPc treatment, B16-F10 were treated and then irradiated for 10 min (660 m, 25.88 J/cm^2^). Day 3: Different analyses were carried out 24 h after the treatment.

Murine melanoma cells (B16-F10) were maintained in DMEM supplemented with 10% (*v*:*v*) FBS and 1% (*v*:*v*) antibiotic solution. Cells were kept in an incubator under a humidified atmosphere with 5% CO_2_ at 37 °C. Cells were cultured for 24 h at an initial concentration of 3 × 10^4^ cells per well (96-well cell culture plate), 7 × 10^4^ (24-well cell culture plate), 1 × 10^5^ (12-well cell culture plate) and 1 × 10^6^ (6-well cell culture), then washed twice with PBS and were exposed to culture medium containing different treatments. Cells were irradiated using a light-emitting diode (LED) system for 10 min, at 10 cm of distance, and with a fluence of 25.88 J/cm^2^. The control (unstimulated) consisted of cells that received only a culture medium. After that, cells were washed with PBS, cultured for a further 15 min, 4 h, or 24 h, depending on the type of analysis to be performed, and finally the analyses were performed (Figure 1).

#### 2.12.1. SLN-AlPc Internalization Assay and Endocytosis Pathway Analysis

The B16-F10 cells were plated in 24-well plates and treated with SLN-AlPc, after 5, 15, and 30 min in dark (no exposure to light), in the presence or not of endocytosis pathway inhibitors (Table 3). After exposure, cells were washed and analyzed using a BD FACSCalibur flow cytometer (BD Biosciences, San Jose, CA, USA) with the FL4 channel. 

#### 2.12.2. Cell Viability and Cell Death Assays

For cell viability quantification, the treated B16-F10 cells were exposed to 3,(4,5-dimethylthiazol-2-yl)-2,5-diphenyltetrazolium bromide (MTT) solution (0.5 mg/mL in culture medium) [19]. Next, the B16-F10 cells were washed with PBS, and the formazan formed by viable cells was extracted with 200 μL DMSO. The absorption at λ 595 nm was then measured using a spectrophotometer (SpectramaxM2; Molecular Devices LLC, San Jose, CA, USA), and the results were expressed as percentages relative to control. Two-way ANOVA was performed with Bonferroni post-test, for comparison between treatments and concentrations at different times.

For the cell viability image, the treated B16-F10 cells were fixed with 4% of paraformaldehyde and stained with 0.05% crystal violet solution for 30 min. Finally, they were washed and measurements at an excitation wavelength of 570 nm using a multimode Microplate Spectrophotometer were taken. For cell death characterization, the treated B16-F10 cells were stained with FITC Annexin V and Propidium Iodide and analyzed using a BD FACSCalibur flow cytometer (BD Biosciences, San Jose, CA, USA).

#### 2.12.3. B16-F10 Cell Morphology

To evaluate the B16-F10 cell morphology, scanning electron microscopy (SEM) and transmission electron microscopy (TEM) were performed as described by da Rocha et al. (2020) [20]. Briefly, for SEM, the cultured B16-F10 cells were fixed using the Karnovsky solution (2% glutaraldehyde, 2% paraformaldehyde, and 3% sucrose in sodium cacodylate buffer 0.1 M pH 7.2) overnight. Then, the cells were washed with sodium cacodylate buffer (0.1 M) and fixed with osmium tetroxide for 30 min. Finally, a sample dehydration gradient of acetone (30–100%) was performed; then, samples were critical-point-dried (CPD 030, Balzers, Germany), gold-sputtered in a SCD metallization (Leica, Wetzlar, Germany), and analyzed in MEV (Jeol 7001 F. Tokyo, Japan). For TEM, B16-F10 cells were fixed using the same protocol as mentioned above. Then, samples were counterstained for 12 h with 0.5% uranyl acetate at 4 °C, and a dehydration gradient of acetone (30–100%) was performed. Finally, the samples were embedded in Epon™ resin, and ultrathin sections (50–70 nm) were obtained with diamond knives in an ultramicrotome (Leica Microsystems, Vienna, Austria). The sections were mounted on copper screens and analyzed using a TEM (Jeol 1011, Tokyo, Japan) operated at an accelerating voltage of 80 kV.

#### 2.12.4. In Vitro ROS Production after SLN-AlPc-PDT

The ROS-sensitive spin probe CMH (stock solution of 10 mM prepared with KHB, which contains 25 μM DF and 5 μM DETC to minimize the oxidation of CMH by Fenton reaction because of transition metals) was added to a final concentration of 250 μM cell culture medium before the irradiation with LED 660 nm. Then, treated B16-F10 were irradiated for PDT. Finally, the supernatant was transferred to a decapped syringe and snap frozen. All the samples were stored at −80 °C until further use. EPR measurements were carried out in a Bruker spectrometer (Bruker EMXplus, Bremen, Germany), equipped with an X-band (9 GHz) high sensitivity cavity (Bruker ER 4119HS, Bremen, Germany). For ROS detection, samples were transferred to liquid nitrogen storage (Noxygen, Bremen Germany), and the spectra were recorded at 77 K. The equipment settings were 2 mW microwave power, 5 G amplitude modulation, 100 kHz modulation frequency, and 200 G sweep width. The peak-to-peak amplitude was employed for the detection of the signal. A calibration curve was obtained using the nitroxide radical (CP·) diluted in KHB to the following concentrations: 0, 10, 50, 100, 250, and 500 μM. In this concentration range, a linear calibration curve was obtained, and all the recorded data were within this calibration range.

#### 2.12.5. Actin Visualization

Briefly, cells were fixed with 3.7% paraformaldehyde for 15 min, washed with phosphate buffer saline (PBS), and permeabilized with 0.1% Triton X-100 for 20 min. B16-F10 cells were incubated with blocking solution (2.5%) containing bovine serum albumin (BSA), and 8% fetal bovine serum and washed. Finally, a conjugate phalloidin-Alexa Fluor^®^ 488 (3:40) and DAPI were added. The slides were mounted with ProLong Gold Antifade, and the images were obtained by confocal microscopy (Leica TCS SP5, Leica Microsystems, Westzlar, Germany).

#### 2.12.6. Western Blotting

Proteins from B16-F10 cell lysate were extracted using lysis buffer (Tris-HCl 50 mM, NaCl 150 mM, EDTA 5 mM, and Triton-X100 1%) and Cocktail Protease Inhibitor (04693159001, Roche, Munich, Germany). The gel contained 12% of polyacrylamide, and the transfer occurred using a dry system. The membrane was blocked for 1 h and incubated overnight at 4 °C with a primary antibody (anti-Bax, B3428—Sigma Aldrich, St. Louis, MO, USA; anti-Bcl2, MA5-11757—ThermoFischer, Waltham, MA, USA; anti-caspase 3, ab13847, Abcam, Cambridge, UK). The membrane was then incubated for 1 h with a rabbit anti-mouse secondary antibody (111-035-006, Jacksons Immuno Research, West Grove, PA, USA). The loading control used was an anti-β-actin antibody (A3854, Aldrich, MO, USA). The bands were revealed using Chemiluminescent Substrate (Westar Supernova XLS3L and XLS3P) by Image Quant LAS 4000 (GE Healthcare Life Sciences, Singapore). The bands were analyzed with the ImageJ software (Version 1.8).

## 3. Results and Discussion

### 3.1. Development and Optimization of Formulations

**Figure 2:**
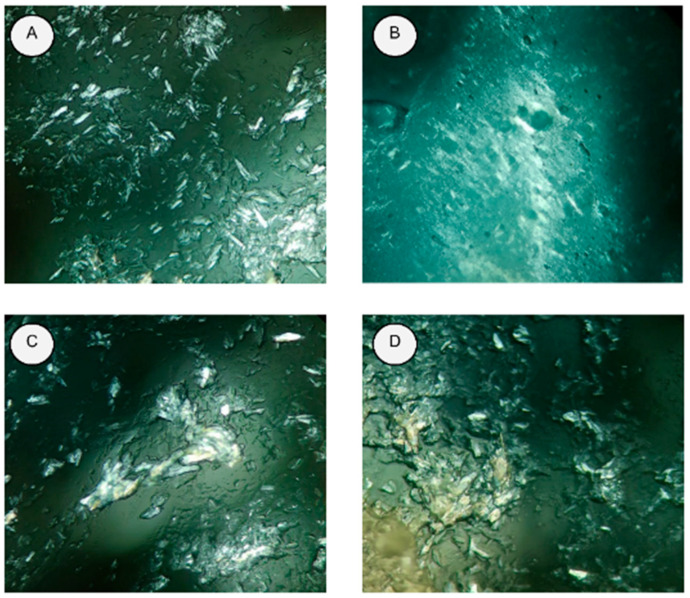
Images obtained with the technique of polarized light microscopy with 40× magnification of the butters: (**A**) murumuru; (**B**) babassu; (**C**) bacuri; (**D**) ucuuba.

Murumuru, babassu, bacuri, and ucuuba butters were all liquid above 70 °C and crystallized after cooling. It is also worth mentioning that babassu butter showed the lowest crystal formation compared to the other samples, a property estimated when forming SLNs, given the stability of more amorphous structures (Figure 2) [21].

Based on the previous data (Table 4), all butters were selected to develop preliminary SLNs. It is suggested that the differences found in HD and PdI between the SLNs formed with different butters stem from the difference in lipid composition, since natural butters are complex mixtures of triacylglycerides whose compositions vary according to the species, and these differences impact their melting point and crystallinity profile [14,22,23]. Thus, the SLN murumuru sample was selected for optimization.

**Figure 3:**
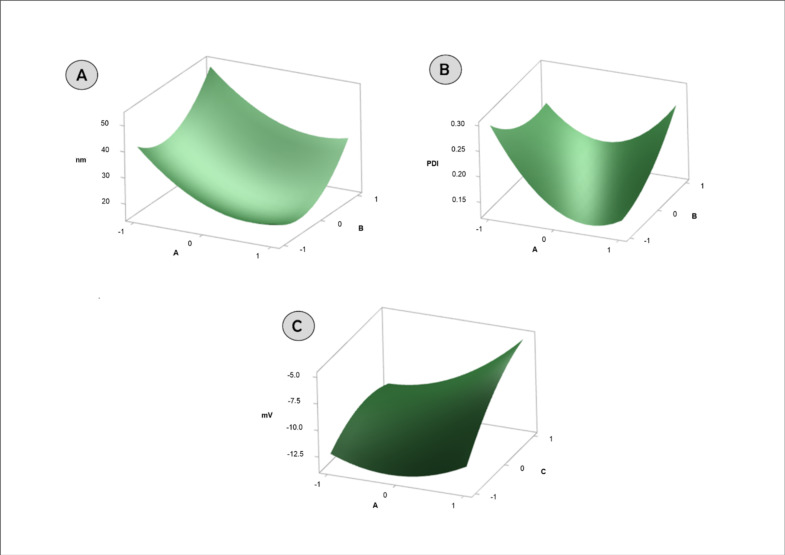
Response surface graphs obtained after BB analysis of the variables: HD (**A**), PdI (**B**), and ZP (**C**).

The HD of the nanoparticles varied between 16.96 nm and 44.52 nm. The value of the correlation coefficient (R2) for Equation (3) was 0.94, indicating a good fit (Figure 3A).

HD = 19.72 *−* 9.61A + 6.35B + 2.50C + 7.91A2 + 9.98B2 + 1.62C2 + 0.59AB *−* 6.66 AC *−* 1.53 BC (3)

The variables A (proportion between surfactant compacting and butter) and B (amount of drug) significantly impacted HD, demonstrating that increasing surfactant concentration promotes diameter reduction and increasing drug concentration promotes an increase in this variable. The reduction of HD with increasing concentration of the surfactant is widely discussed in the literature, being related to the disruption of surface tension and increased ability to cover lipid droplets [24].

The PdI of the nanoparticles varied between 0.125 and 0.315. The value of the correlation coefficient (R2) for Equation (4) was 0.97, indicating a good fit (Figure 3B).

PdI = 0.1377 *−* 0.027A + 0.0131B *−* 0.0152C + 0.074A2 + 0.0249B2 + 0.0182C2 + 0.0455AB *−* 0.0483 AC *−* 0.0048 BC (4)

Considering that the ratio between surfactant and butter significantly impacted the PdI, there is an optimal region for the amount of surfactant which promotes the development of SLN with reduced PdI. Thus, it is suggested that low concentrations of surfactant are not capable of forming monodisperse structures, and that excess surfactant is capable of forming differentiated structures such as micelles, leading to the formation of polydisperse nanosuspensions.

The ZP of the nanoparticles varied between −13.30 mV and −4.37. The value of the correlation coefficient (R2) for Equation (5) was 0.93, indicating a good fit (Figure 3C).

ZP = −10.423 + 1.759A + 0.834B + 1.922C + 1.497A2 + 0.797B2 − 1.126C2 − 0.555AB + 1.348 AC + 0.573 BC(5)

Considering that the proportion of variables between surfactant and butter and the temperature significantly impacted the ZP, it is observed that the increase in surfactant concentration and formation temperature promoted the development of SLN with PZ with less negative charges. The increase in charges proportional to the increase in surfactant concentration is due to its anionic character. It is suggested that higher temperatures promote better covering capacity for the surfactant.

Thus, considering the aforementioned aspects, the selected SLN was composed of the 2:1 ratio (surfactant and murumuru butter), 20 μM of AlPc, and heating at 85 °C. The optimization yielded lower HD, more monodisperse nanosuspensions, increased PZ, and increased EE% of AlPc (Table 5). The reduction of HD is associated with greater adhesion of nanoparticles to the skin and may optimize therapy [25,26]. As mentioned before, PdI is useful to predict the colloidal stability, so the optimization promoted the formation of a more monodisperse nanosuspension, with possible colloidal stability superior to that previously developed.

The increase in PZ is related to the increase in the concentration of non-ionic surfactant used, which can also influence the increase in the observed EE%, since the surfactant can suppress the formation of crystalline events [27].

### 3.2. Differential Scanning Calorimetry

The developed SLNs had a higher melting point (>90 °C) than the butter alone (38.37 °C), suggesting the formation of systems with a higher degree of organization for the murumuru butter (Table 6). The higher enthalpy variation in the SLNs is related to the large surface area per volume demonstrated by these systems [20], justifying the greater variation between the nanosystems in relation to butter and the drug alone. The presence of AlPc in the nanoparticle reduced the melting point of the nanosystem, suggesting that that placement of the PS molecules among the lipid carbon chains could reduce the butter’ crystaline organization after cooling. Additionally, the absence of the endothermic event characteristic of AlPc was observed in the thermogram of the SLN sample, suggesting that it is molecularly dispersed in the nanostructure [16,28].

### 3.3. In Vitro Skin Permeation

**Figure 4:**
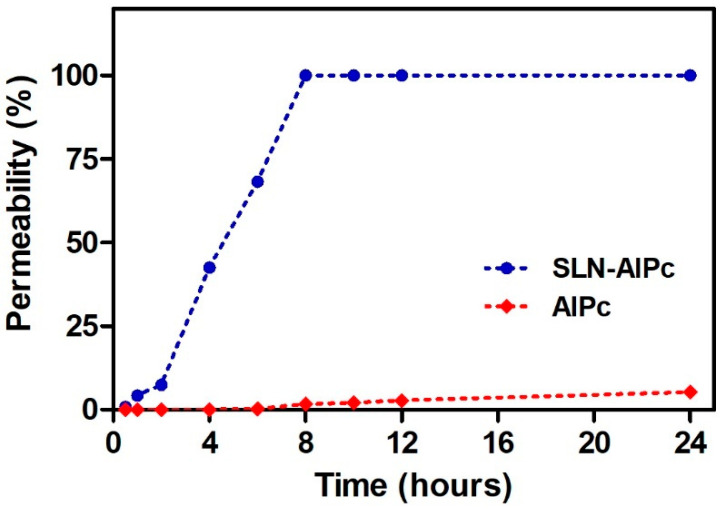
In vitro skin permeation profile of AlPc and SLN-AlPc over 24 h.

The quantification of permeated samples was performed in a spectrofluorometer, due to the low concentration of AlPc that permeated being below the limit of quantification of HPLC, as also demonstrated in the works of De Souza et al. (2016) [29] and Silva et al. (2013) [30]. In Figure 4, it is possible to verify that AlPc in solution did not permeate the skin; similarly, the permeation of AlPc was not verified by Almeida et al. (2018) [31], as it was retained in the stratum corneum. However, when encapsulated in SLN, it was possible to verify a permeation of practically 100% with 8 h of contact. The total permeation of SLN-AlPc may have been influenced by its low hydrodynamic diameter (~17 nm) [32], allowing skin permeation, different from what was observed by Almeida et al. (2018) [26], who evaluated SLN loaded with AlPc with particle size greater than 200 nm, where there was no permeation in 24 h of analysis, only penetration in the dermis and epidermis. In addition to the small size, which facilitates the influx of the drug through the skin, the ability of SLN to adhere to the stratum corneum allows greater penetration of the encapsulated sample [33,34].

### 3.4. Morphology of SLN and SLN-AlPc

**Figure 5:**
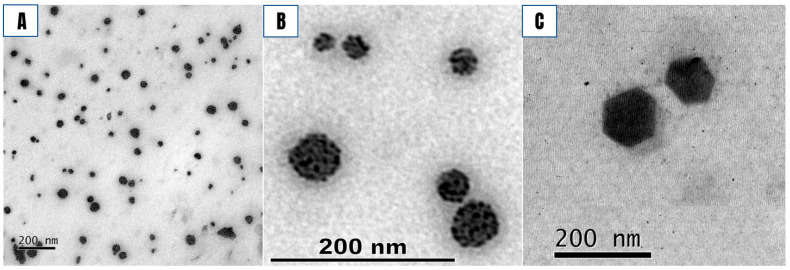
Transmission electron microscopy images of SLN-AlPc (**A**,**B**) and SLN (**C**). The micrographs show a spherical shape with small dark spots (**A**,**B**), suggesting the presence of AlPc. AlPc has a high affinity for osmium tetroxide, which explains the darker labeling. It is possible to observe that the SLN (**C**) presents a different morphology.

The SLNs presented a polyhedric morphology (Figure 5C), while SLN-AlPc showed a rounded shape with small dark spots (Figure 5A,B). The hypothesis for the data found in the present work is that AlPc, which is extremely hydrophobic, has physically interacted with the mixture of lipid and surfactant, preventing the formation of crystals during the cooling of the formulation. In summary, the finding showed that the crystallization and polymorphic transformation of the oil phase can be modified by the interaction of AlPc with the surface of crystals. This result can be useful in the application of emulsions that aim to improve the solubility and release profile of phthalocyanines [35,36,37,38].

### 3.5. Spectroscopic Analysis

The FTIR spectra of the SLN-AlPc and SLN formulations (Figure S1a,b, respectively) show typical features of lipids, especially murumuru butter (Figure S1c), in agreement with Ganassin et al. (2022) [27]. The FTIR spectrum of AlPc free is showed in Figure S1d. Direct evidence of the presence of AlPc is not observed in the spectra of the SLN-AlPc formulations. Although very similar, the FTIR spectra of SLN and SLN-AlPc formulations present small differences. The main spectral differences are observed in the regions from 1000–1150 cm^−1^, 1700–1800 cm^−1^, and 2850–3000 cm^−1^, which are assigned to the stretching vibration of the C-O and C=O, the lipid ester carbonyl groups, and CH_2_ groups of the hydrocarbon chains, respectively. A comparison between the IR absorption spectra of the SLN and SLN-AlPc formulations shows that, after loading with AlPc, the C=O and ν_s_(CH_2_), ν_as_(CH_2_) modes become narrower and redshifted when compared to vibrational modes from the SLN formulation. Decreases in the frequency and bandwidth of the ν(C=O) of the ν_s_(CH_2_), ν_as_(CH_2_) modes are related to the changes in the polarity of the local environment around the ester carbonyl group and the increase in the hydrocarbon chain conformational order [39]. Thus, the spectral changes observed suggest that the presence of AlPc is stabilizing the system through interactions of the AlPc molecule with the ester carbonyl groups of the lipid hydrocarbon chains.

The hypothesis reported above is supported by data of normal Raman spectroscopy. As with the IR absorption spectrum, the normal Raman spectra of the SLN-AlPc and SLN formulations, displayed in Figure S2a,b, show similar features to the normal Raman spectrum of murumuru butter (Figure S2c). The normal Raman spectrum of AlPc free is showed in Figure S1d. Again, no direct evidence of the presence of AlPc is observed in the Raman spectrum of the SLN-AlPc formulation. However, spectral changes are observed that support the changes in the polarity of the local environment around the ester carbonyl group and the increased conformational order in the hydrocarbon chains. For example, the changes observed in the regions from 1000–1150 cm^−1^ and 1400–1500 cm^−1^ are diagnostic of improved alkyl chain packing arrangements, as they reflect concomitant increases in both hydrocarbon chain mobility and the relative content of trans/gauche conformers [40].

**Figure 6:**
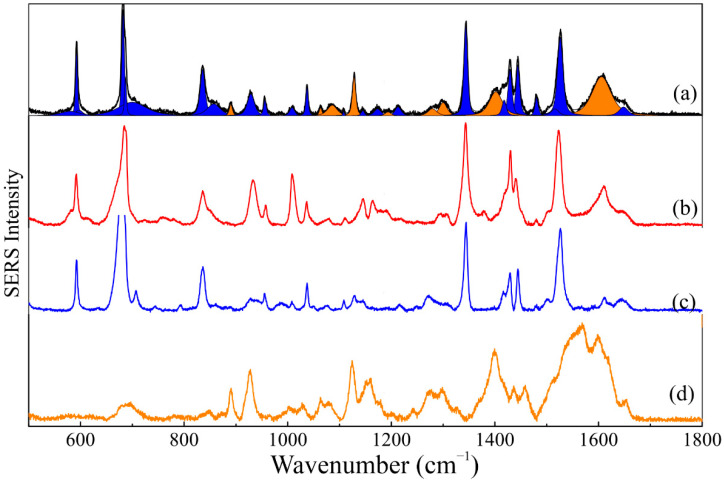
SERS spectra of (**a**) SLN-AlPc formulation, (**b**) physical mixture (AlPc + SLN), (**c**) solution of AlPc in alcohol, and (**d**) SLN solution of AlPc in alcohol.

Unlike FTIR and normal Raman spectroscopy, the SERS technique proved to be adequate to show the vibrational modes of both components of the SLN-AlPc formulation. The SERS spectrum of the SLN-AlPc formulation, shown in Figure 6a, appears to be an overlap of the SERS spectra of each component individually (Figure 6c,d for AlPc free and SLN components, respectively). Thus, to investigate this behavior, a physical mixture of AlPc with SLN formation (AlPc + SLN) was prepared, and the SERS spectrum of this solution was recorded (Figure 6b) and compared with the SERS spectrum of the SLN-AlPc formulation. A careful analysis shows important differences between the SERS spectra of the SLN-AlPc formulation and the AlPc + SLN physical mixture (see Figure S3—SLN-AlPc: black lines and AlPc + SL: red lines).

Actually, a careful evaluation of the SER spectra of both the SLN-AlPc formulation and the AlPc + SLN sample evidences that while the vibrational energies associated with the macroring (at ~590, 830, 1340, and 1430 cm^−1^) and benzene groups (at ~1030 and 1480 cm^−1^) remain constant in both samples, the vibrational modes associated with the isoindole groups (680 and 960 cm^−1^) and the C-N-C and C-N bonds (at ~1445 and 1520 cm^−1^, respectively) in the latter present considerable and systematic blueshift with respect to the former [17,41].

Based on the evidence above, it is not possible to state that the AlPc molecules interact directly with the hydrocarbon chains of murumuru butter. Most likely, the Raman shifts observed in the SERS spectrum of the physical mixture are associated with the interaction of the AlPc molecule with the silver substrate, used in the SERS experiment, via the nitrogen of the isoindole groups of the AlPc molecule. In the SLN-AlPc formulation, the AlPc molecules do not form a direct bond with the SLN nanoparticles. Moreover, the AlPc are not adsorbed onto the silver film’s surface, likely due to steric hindrance promoted by the murumuru batter template. Nevertheless, the presence of the AlPc vibrational modes in the SER spectrum shows that, even without surface adsorption, the SERS effect indeed occurs. Finally, vibrational spectroscopy data indicate the AlPc molecules were loaded successfully onto the SLN formulation structure.

### 3.6. Photophysical Properties

It was found that SLN-AlPc containing final AlPc concentrations of 20 μM and 40 μM (SLN-AlPc-20 and SLN-AlPc-40, respectively) presented absorbance peaks of 676 nm and 674 nm, respectively (Figure S4). This is an important characteristic to be considered when evaluating its use in anticancer PDT, since the ideal therapeutic window for PDT is in the spectral region from 600–800 nm, where radiation penetration into living tissue is greater and absorption by endogenous chromophores (water, proteins, and pigments such as melanin and bilirubin) is lower [42,43].

### 3.7. Stability under Storage

During the 176 days of observation, the three nanosystems remained stable at RT (Room Temperature) and 4 °C, with no significant change (*p* < 0.05) in DH, PDI, and PZ (Table S1). These results are in accordance with other studies from our group, confirming the reproducibility of this protocol [20]. 

### 3.8. Internalization, Mechanisms of Endocytosis, and Subcellular Location of SLN-AlPc

**Figure 7:**
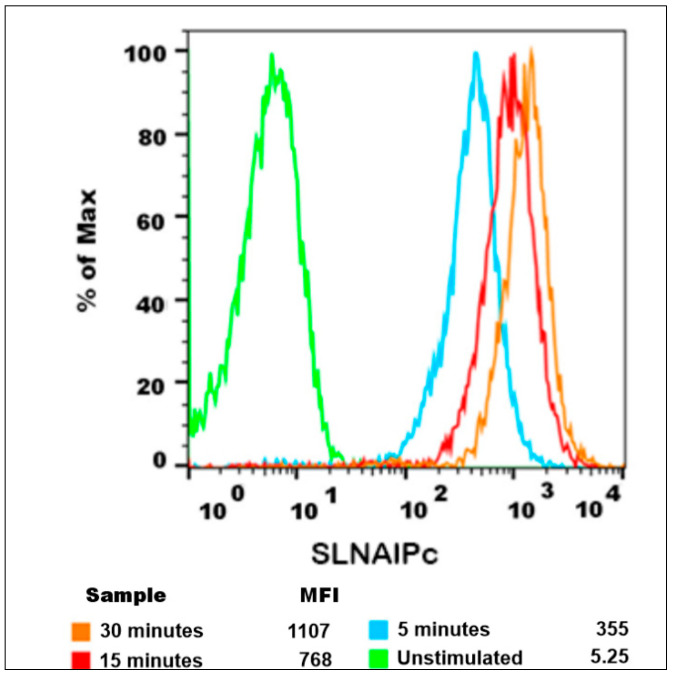
Internalization of SLN-AlPc. The fluorescence histogram showed the internalized amount of SLN-AlPc at different time points after the SLN-AlPc treatment. The fluorescence signal increases proportionally to the time that the B16-F10 were exposed to SLN-AlPc.

**Figure 8:**
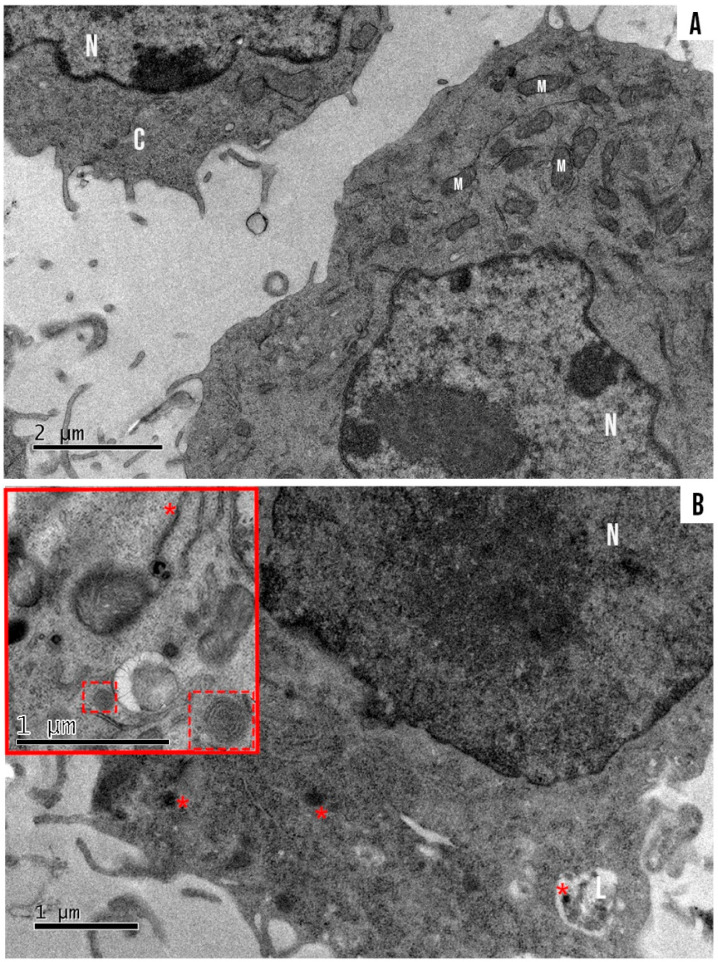
(**A**) Ultrastructure of B16-F10 treated with SLN-AlPc for 15 min. (**B**) Internalized aggregated SLN-AlPc is present in B16-F10 (*) and in a liposome (L). Melanosoma is present in the melanocyte cells (slashed box). Nucleus (N), mitochondria (M), and cytoplasm (C) are indicated in the image.

B16-F10 cells exposed for 15 and 30 min showed higher AlPc-specific fluorescence intensities than at 5 min (Figure 7). The highest fluorescence intensity was observed at 30 min (orange peak) of exposure. It is possible to observe in the histograms of fluorescence intensity that the peak area related to the time of 15 min covers most areas of the peaks of 5 min and 15 min (red peak). Therefore, the time chosen for the next experiments was 15 min. A reduced AlPc exposure time, in addition to decreasing possible toxicity in non-irradiated groups, also facilitated multi-step protocols and experiments. The 15 min time point of exposure before irradiation was also used in other publications by our group [17,44,45]. The internalization of SLN-AlPc, as well as its interaction with organelles of the B16-F10 cell lineage, could also be observed by TEM (Figure 8).

**Figure 9:**
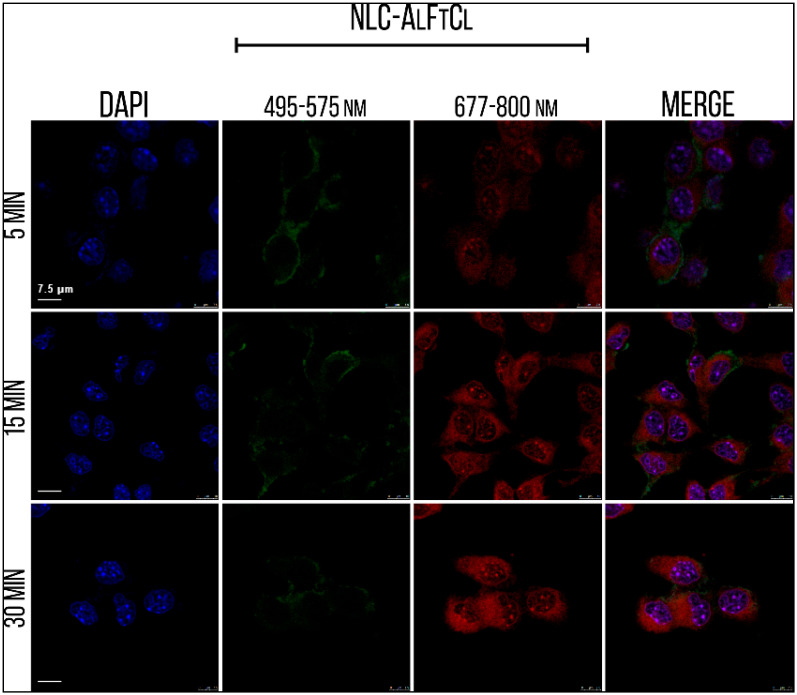
Internalization of SLN-AlPc after 5, 15, and 30 min of treatment. The nucleus (blue), the SLN-AlPc (green), and the AlPc (red) are demonstrated in the image.

During the analysis of SLN-AlPc internalization by confocal laser fluorescence microscopy, it was noted that the AlPc (red) was already colocalized with the nucleus labeled with DAPI (blue) in B16-F10 cells just 15 min after the treatment (Figure 9).

**Figure 10:**
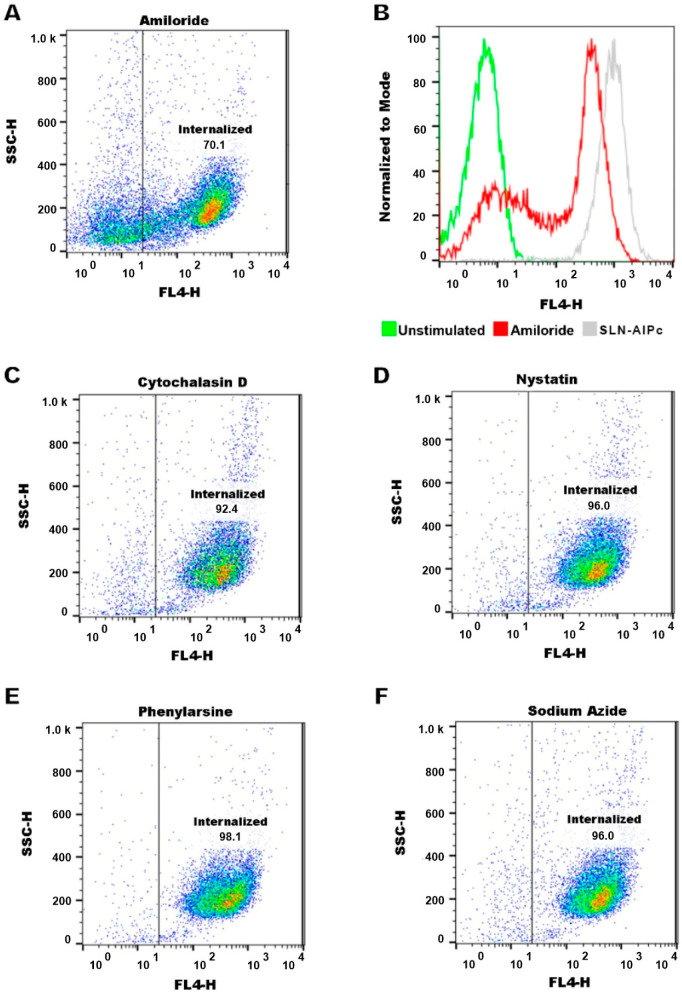
Endocytosis pathway characterization using different endocytosis inhibitors: (**A**) a reduction in the fluorescence signal of SLN-AlPc was observed when treated with amiloride, indicating a decrease in SLN-AlPc internalization by the macropinocytosis pathway; (**B**) histogram of the fluorescence signal in B16-F10 cells treated with amiloride and SLN-AlPc. The cytochalasin D (**C**), nystatin (**D**), phenylarsin (**E**), and sodium azide (**F**) did not alter the fluorescence signal of SLN-AlPc.

**Figure 11:**
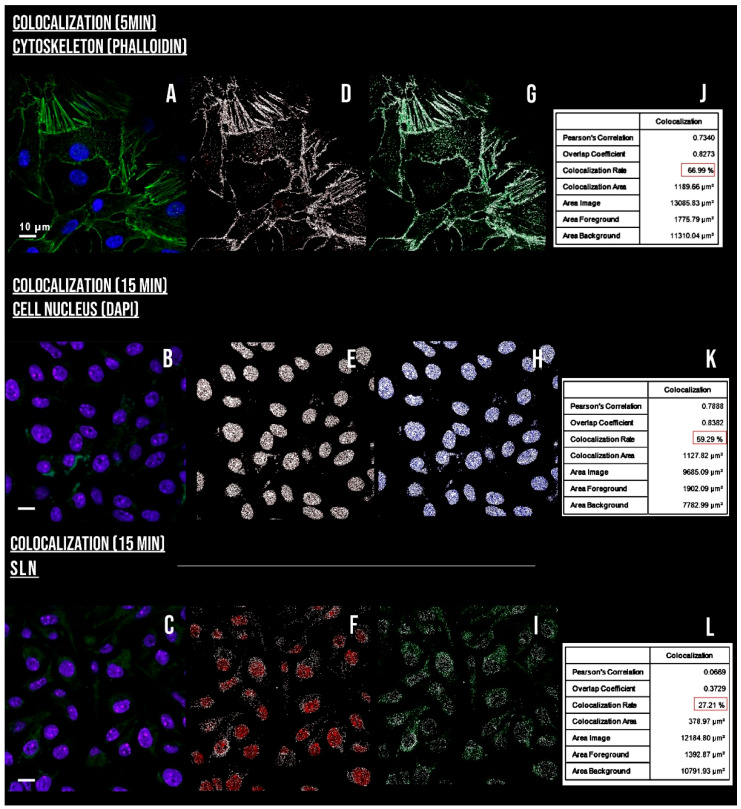
Colocalization assays of SLN-AlPc. The figure shows the colocalization of fluorescence relative to AlPc for cytoskeleton (**A**,**D**,**G**,**J**), nucleus (**B**,**E**,**H**,**K**), and fluorescence relative to SLN-AlPc (**C**,**F**,**I**,**L**). The white dots in the figures represent positive fluorescence pixels, indicating colocalization. In the last column, the colocalization index is shown in %, in addition to the Pearson coefficient that was used for the analyses.

**Figure 12:**
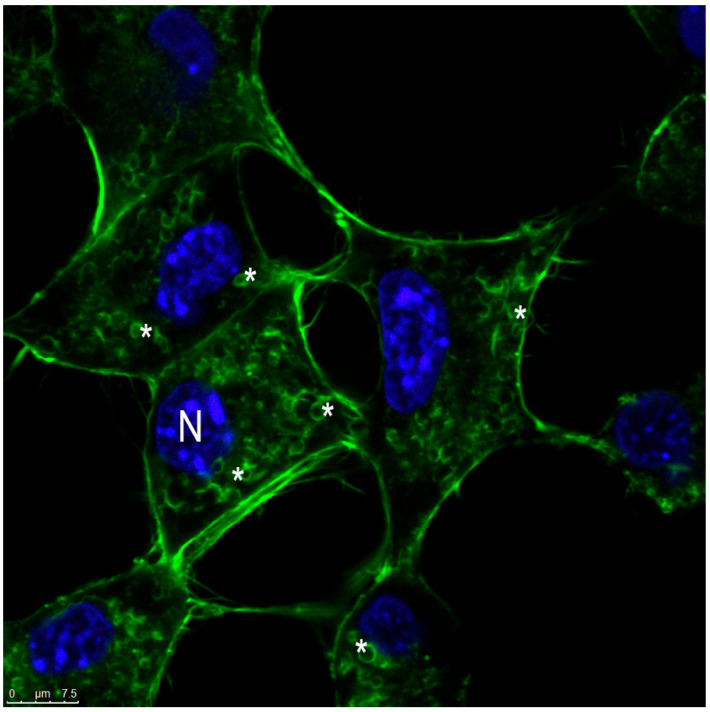
The cytoskeleton of B16-F10 cells. Macropynosomes (*) can be observed in B16-F10 cells treated with SLN-AlPc. In blue, the nucleus (N) can be observed.

It was observed that only cells treated with the inhibitor amiloride showed a decreased AlPc-specific fluorescence intensity, indicated by a shift to the left, which is indicated in Figure 10. This shows that macropinocytosis maybe the main contributor to the nanocarrier internalization. This pathway is characterized by the movement of the plasma membrane induced by a global activation of the actin cytoskeleton. This pathway involves the transposition of a large amount of external colloid through the formation of ripples along the plasma membrane. Cytoplasmic projections merged into the membrane as waves, resulting in large endocytic vacuoles, heterogeneous in size and morphology, called macropinosomes [46]. The colocalization of SLN-AlPc with the cytoskeleton (Figure 11) and the visualization of spherical actin structures after exposure of B16-F10 cells to the carrier (Figure 12), suggesting macropinosomes, confirmed the occurrence of endocytosis of SLN-AlPc mediated by macropinocytosis.

### 3.9. In Vitro Tests for the Assessment of Safety, Effectiveness, and Cell Death Analyses

**Figure 13:**
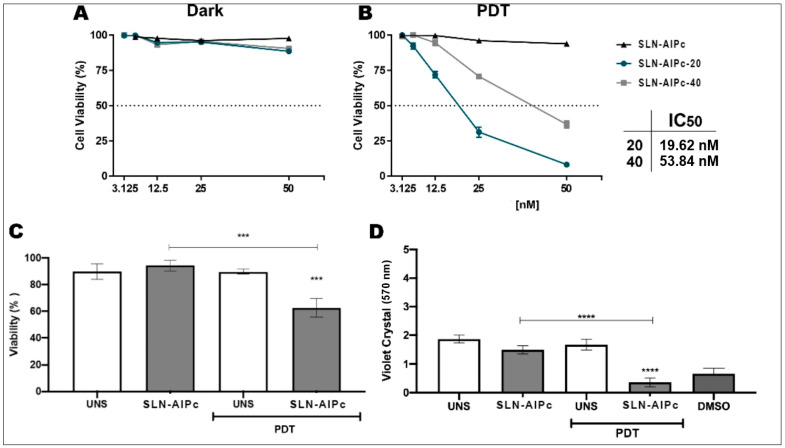
Cellular viability of B16-F10 cells treated with SLN-AlPc. The percentage of B16-F10 cell viability was calculated at different concentrations of SLN-AlPc treatment (**A**,**B**). The percentage of B16-F10 cell viability was calculated at different treatment conditions using propidium iodide (**C**). The absorbance of violet crystal was calculated under different treatment conditions to evaluate the cell death (**D**) *** *p* < 0.001 and **** *p* < 0.0001 compared to control.

When B16-F10 cells were exposed to the SLN-AlPc and irradiated (Figure 13B), a significant decrease in viability of B16-F10 cells was observed, with an inhibitory concentration (cytotoxic) of 50% (IC_50_) of 19.62 nM. As for SLN-AlPc-40, the IC_50_ value was 53.84 nM. As expected, SLN-AlPc was innocuous in the dark. The results corroborate other works that indicate that PDT is minimally invasive and, when used with light and a photosensitizer to selectively target cancer cells, can minimize effects to surrounding healthy tissues [10]. In addition to the MTT test, the crystal violet test (Figure 13D) and cell death analyses by flow cytometry (Figure 13C) were used to evaluate cell viability. The LED light alone, the blank SLN, and the SLN-AlPc in the dark (Figure 13A) did not affect the viability of the cells.

SLN-AlPc-20 (20 μM) showed better photodynamic activity than SLN-AlPc-40 (40 μM) during the PDT (Figure 13B). This behavior can be explained by the fluorescence the formation of aggregates of AlPc, leading to low fluorescence emission suppression by quenching phenomena, by close proximity of the photosensitizing molecules [17].

**Figure 14:**
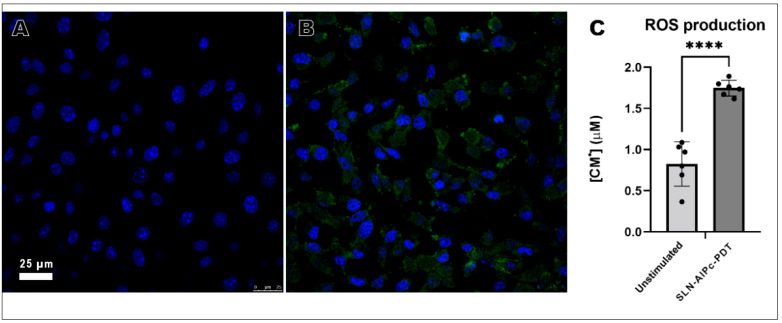
ROS production in B16-F10 cells treated with SLN-AlPc. Images were taken in confocal microscopy (**A**,**B**) and paramagnetic resonance (**C**). (**A**) B16-F10 cells that were not irradiated had no ROS production (green) since only the nucleus (blue) could be seen in the image. (**B**) ROS production was observed after B16-F10 irradiation. (**C**) Quantification of ROS production in different treatment conditions. **** *p* < 0.0001 compared to control.

B16-F10 cells had a high capacity to produce ROS, during treatment (Figure 14).

### 3.10. SLN-AlPc-PDT Induces Apoptosis

**Figure 15:**
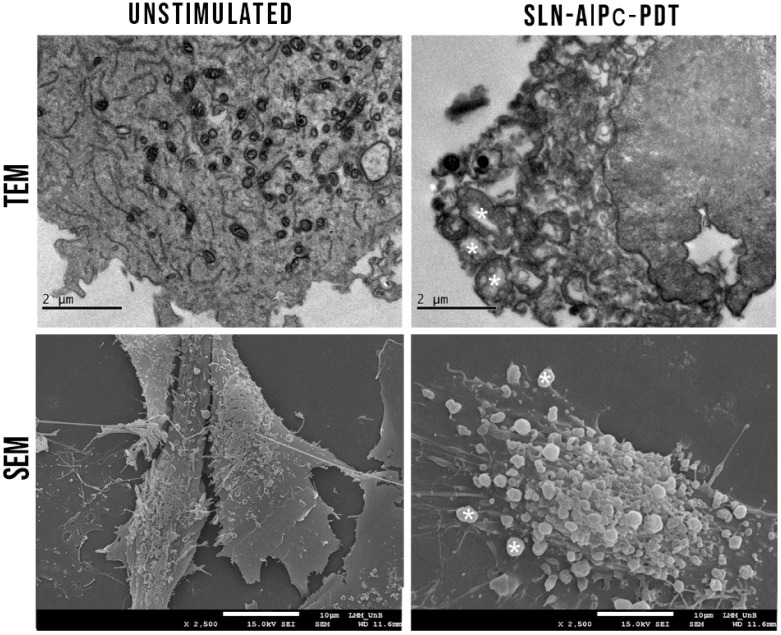
B16-F10 cells morphology in TEM and SEM after the PDT treatment with SLN-AlPc; it was also possible to observe formation of apoptotic bodies (*) and cytoplasmic bumps.

Regarding B16-F10 cell morphology, it was also possible to observe the formation of apoptotic bodies and cytoplasmic bumps (Figure 15). This result is further evaluated to determine which type of cell death the PDT treatment is inducing in B16-F10 cells.

**Figure 16:**
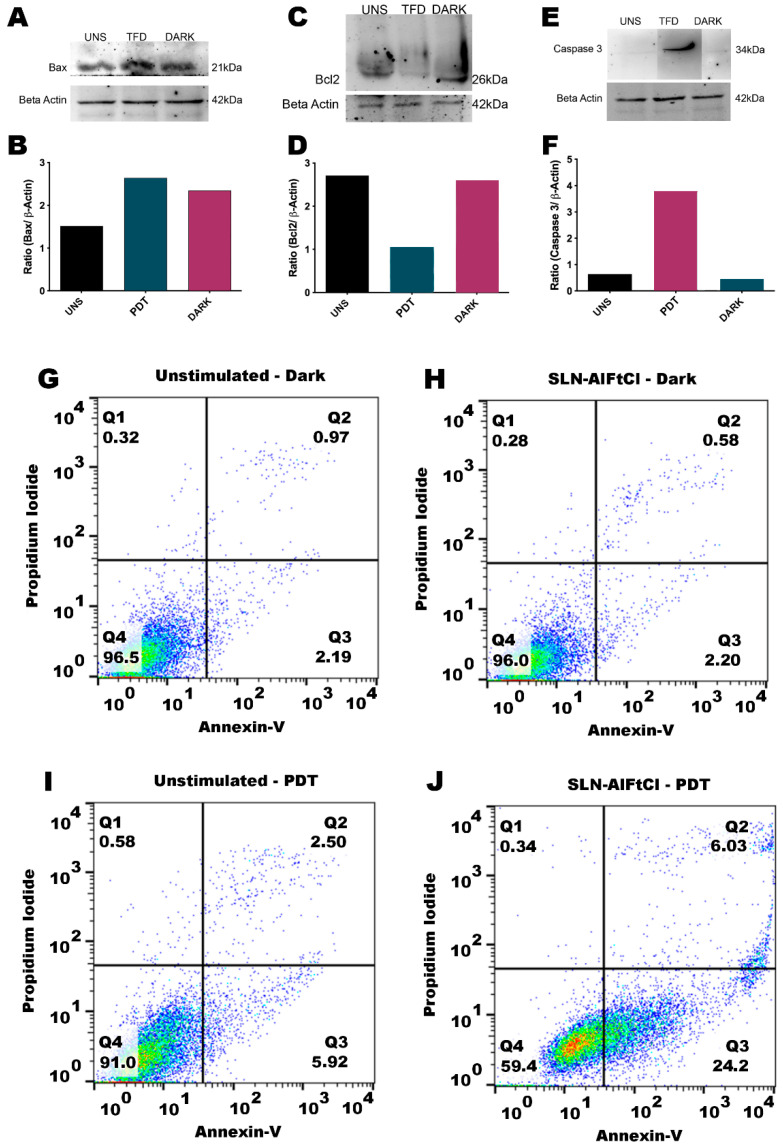
Analysis of apoptotic cell death. Apoptosis was evaluated by Western blot (**A**–**F**) and flow cytometry (**G**–**J**). Western blot images of Bax (**A**), Bcl2 (**C**), and caspase 3 (**E**) bands as well as the loading control (beta acting) bands. Pixel quantification of Bax (**B**), Bcl2 (**D**), and caspase 3 (**F**) bands to the loading control bands. Apoptosis was induced by phosphatidylserine under different treatment conditions (**G**–**J**). Q1 represents the Propidium Iodine positive signal, which is a marker for necrosis. Q2 represents both propidium iodide (necrose) and Annexin-V (Apoptose) positive signal. Q3 represents only Annexin-V positive signal, and Q4 represents no positive signal and, therefore, no cell death.

In the present study, the exposed phosphatidylserine was identified on the outer leaflet of the plasma membrane of B16-F10 cells submitted to the PDT (Figure 16J), which evidences that cells undergo apoptosis after this treatment. To confirm and detail the type of death caused by the proposed treatment, Bax (Figure 16A,B), Bcl2 (Figure 16C,D), and Caspase 3 (Figure 16E,F) proteins were quantified by Western blotting. Pixel quantification of Bax (Figure 16B), Bcl2 (Figure 16D), and caspase 3 (Figure 16G) bands to the loading control bands. Apoptosis is induced by phosphatidylserine under different treatment conditions (Figure 16G–J).

Treatment with SLN-AlPc-PDT triggered a high expression of caspase-3 and a lower expression of Bcl2. According to Garg et al. (2014) [47,48], PDT can promote the activation of death by apoptosis through the activation of caspases—cysteine-dependent aspartate-specific proteases—and decrease the expression of apoptosis-inhibiting proteins, such as Bcl-2 [17,49].

## 4. Conclusions

This work reported the development of a solid lipid nanoparticle containing AlPc, a hydrophobic phthalocyanine derivative. This formulation showed photodynamic activity in aqueous media, the distribution of hydrophobic drugs in aqueous media is an advantage for the routes of administration in patients and association with other treatments and thus was effective in reducing the viability of B16-F10 cells in vitro. The treatment generates reactive oxygen species, overcoming the antioxidant action of melanin produced by the cell, causing relevant cell damage. The main type of cell death caused by the treatment is apoptosis. The present study indicates that the developed lipid nanoparticle is a potential system to transport photosensitizers (PS). By improving water solubility, SLN-AlPc proved to be a great third-generation PS, due to its unique structure. As there is no lipid crystallization, AlPc remains incorporated into the amorphous matrix, offering greater PS charge and increasing its stability during storage. SLN-AlPc is stable and scalable and has the potential for incorporation of photosensitizing molecules and other chemotherapeutics.

## Figures and Tables

**Table 1:** Independent variables selected for Box–Behnken method.

Independent Variables		Levels	
	−1	0	+1
A: Butter: Surfactant	1:1	1:1.5	1:2
B: AlPc (μM)	20	40	80
C: Temperature (°C)	75	80	85

**Table 2:** Combinations generated from independent variables.

Number of Experiment	A	B	C
1	0	1	−1
2	−1	0	1
3	−1	−1	0
4	0	−1	−1
5	0	0	0
6	0	0	0
7	1	0	1
8	−1	0	−1
9	0	−1	1
10	−1	1	0
11	1	−1	0
12	1	1	0
13	0	0	0
14	1	0	−1
15	0	1	1

**Table 3:** Endocytosis inhibitors and their respective target pathways.

Inhibitor	Concentration	Inhibition Pathway
Sodium azid	100 mM	ATP-dependent pathways
Amiloride	0.2 mM	Macropinocytosis
Cytochalasin D	1 μM	Pinocytosis
Phenylarsine	0.2 μM	Clathrin-mediated pathway
Nystatin	20 μg/mL	Caveolin-mediated pathway

**Table 4:** HD, PdI, and ZP obtained with previous SLNs.

Sample	HD (nm)	PdI	ZP (mV)
SLN Murumuru	27.5 ± 0.2	0.191 ± 0.001	−11.80 ± 2.60
SLN Babaçu	100.6 ± 0.2	0.266 ± 0.005	−10.40 ± 0.15
SLN Bacuri	536.4 ± 49.8	0.600 ± 0.039	−22.30 ± 1.18
SLN Ucuuba	140.8 ± 2.7	0.526 ± 0.006	−21.60 ± 0.12

**Table 5:** HD, PdI, ZP, and EE% values before and after BB optimization.

Sample	HD (nm)	PdI	ZP (mV)	EE%
SLN	55.53 ± 0.23	0.191 ± 0.001	−11.80 ± 2.60	61.50 ± 0.67
SLN optimized	17.64 ± 0.21	0.173 ± 0.013	−5.61 ± 0.93	66.40 ± 1.12

**Table 6:** Results were obtained with DSC analysis of murumuru butter, AlPc, and SLN.

Sample	Melting Point (°C)	ΔH (Jg^−1^)
Murumuru butter	38.4	63.9
AlPc	175.1	194.3
SLN	96.3	1036.0
SLN-AlPc	93.3	873.2

## Data Availability

Not applicable.

## Supplementary Materials

The following supporting information can be downloaded at: https://www.mdpi.com/article/10.3390/nano12203547/s1, Figure S1: FTIR spectra of the (a) SLN-AlPc and (b) SLN formulation, (c) murumuru butter and (d) AlPc free; Figure S2: Normal Raman spectra of the (a) SLN-AlPc formulation and (b) SLN formulation, (c) murumuru butter and (d) AlPc free. Figure S3: Zoom, at different spectral ranges, of the SERS spectra of SLN-AlPc formulation (black lines) and physical mixture (AlPc + SLN), (red lines) of samples (i) Gly-solution (ii) 2Gly@PEG/NPs, and (iv) normal Raman spectrum of Gly-solution. Figure S4: Fluorescence (A e B) and absorbance (C) spectrum of SLN-AlPc. A = Ex:660 nm and B = Ex:350 nm. Table S1: Stability of SLN-AlPc by the parameters of DH, IPD and PZ, for 1, 90 and 176 days, at room temperature, 4 °C and 37 °C.
