# Supplementary material for: Development of New Natural Lipid-Based Nanoparticles Loaded with Aluminum-Phthalocyanine for Photodynamic Therapy against Melanoma
Source: Nanomaterials (Basel). 2022 Oct 11;12(20):3547. doi: 10.3390/nano12203547 (PMC9609910; doi:10.3390/nano12203547)
Supplement: Supplementary file 1 [file nanomaterials-12-03547-s001.zip › nanomaterials-1946038-supplementary.pdf]

Development of New Natural Lipid-Based Nanoparticles Loaded with Aluminum-Phthalocyanine for Photodynamic Therapy Against Melanoma

Victor Carlos Mello [1,2,3], Victor Hugo Sousa Araújo [4], Karen Letycia Rodrigues de Paiva [2,3], Marina Mesquita Simões [2,3], Dafne Caroline Marques [2,3], Nelice Roberta da Silva Costa [5], Isadora Florêncio de Souza [2], Patricia Bento da Silva [5], Igor Santos [6], Raquel Almeida [6], Kelly Grace Magalhães [6], Sebastião William da Silva [7], Alexandre Silva Santos[7], Fabiane Veiga-Souza [8,10], Paulo Eduardo Narcizo Souza [9], Marina Arantes Raddichi [2,5], João Paulo Figueiró Longo [5], Jennifer Thayanne Cavalcante de Araújo [4], Luis Alexandre Muehlmann [10], Marlus Chorilli [4] and Sônia Nair Báo [2,*]

1 Postgraduate Program in Animal Biology, Institute of Biological Sciences, Department of Genetics and Morphology, Institute of Biological Sciences, University of Brasília, Brasília, DF 70910-900, Brazil
2 Laboratory of Microscopy and Microanalysis, Department of Cell Biology, Institute of Biological Sciences, University of Brasília, Brasília, DF 70910-900, Brazil
3 COOIL Institute, Brasília, DF 72622401, Brazil
4 School of Pharmaceutical Sciences, São Paulo State University, Araraquara,14800-903, Brazil
5 Laboratory of Nanobiotechnology, Department of Genetics and Morphology, Institute of Biological Sciences, University of Brasilia, Brasilia 70910-900 , Brazil
6 Laboratory of Immunology and Inflammation, Department of Cell Biology, University of Brasilia, Brasilia, DF 70910-900, Brazil
7 Optical Spectroscopy Laboratory, Institute of Physics, University of Brasilia, Brasilia, DF 70910-900, Brazil
8 Laboratory of Protein Chemistry and Biochemistry, Department of Cell Biology, Institute of Biology, University of Brasília, Brasília, DF 70910-900, Brazil
9 Laboratory of Electron Paramagnetic Resonance, Institute of Physics, University of Brasília, Brasília, DF 70910-900, Brazil
10 Faculty of Ceilandia, University of Brasilia, Brasilia, DF 70910-900, Brazil
* Correspondence: snbao@unb.br

Table S1. Stability of SLN-AlPc by the parameters of DH, PDI and PZ, for 1, 90 and 176 days, at room temperature, 4°C and 37°C

	Day 1	Day 90	Day 176
		Room Temperature	
DH (nm)	35.64 ± 0.913	57.53 ± 1.195	60.41 ± 3.41
ZP (mV)	−5.61 ± 0.925	−6.63 ± 0.249	−5.09 ± 0.732
PdI	0.123 ± 0.013	0.118 ± 0.009	0.192 ± 0.027
		4°C	
DH (nm)	35.64 ± 0.913	54.36 ± 1.628	49.12 ± 2.198
ZP (mV)	−5.61 ± 0.925	−7.64 ± 0.826	−5.89 ± 1.012
PdI	0.123 ± 0.013	0.149 ± 0.018	0.192 ± 0.027
		37°C	
DH (nm)	35.64 ± 0.913	257.3 ± 24.60	
ZP (mV)	−5.61 ± 0.925	−6.63 ± 0.249	Phase separation
PdI	0.123 ± 0.013	0.346 ± 0.084	

DH: Hydrodynamic diameter; PDI: Polydispersity index; and PZ: Potential zeta

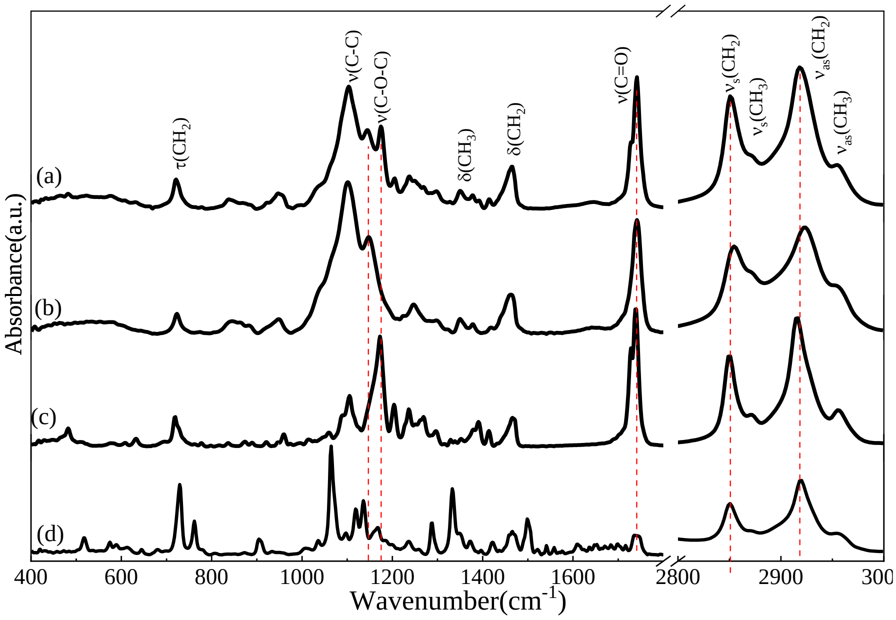

Figure S1. FTIR spectra of the (**a**) SLN-AlPc and (**b**) SLN formulation, (**c**) murumuru butter and (**d**) AlPc free

.

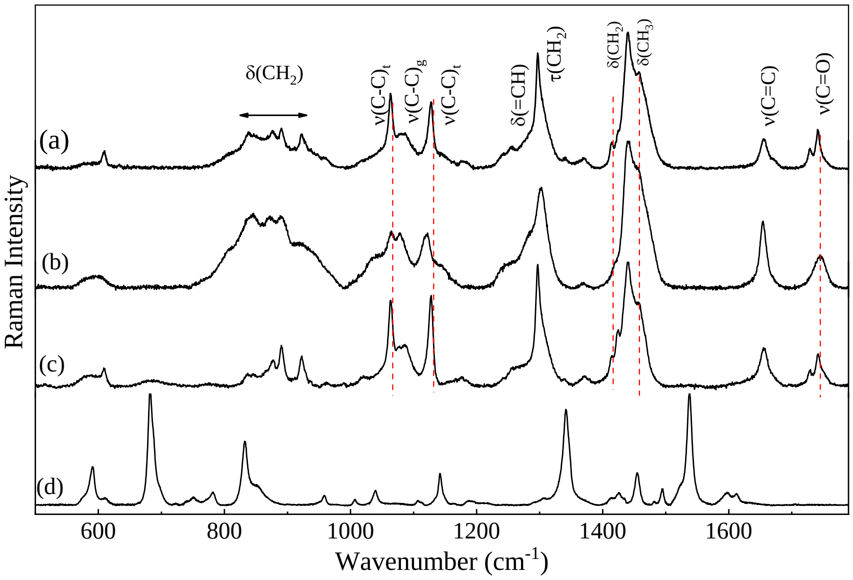

Figure S2. Normal Raman spectra of the (**a**) SLN-AlPc formulation and (**b**) SLN formulation, (**c**) murumuru butter and (**d**) AlPc free.

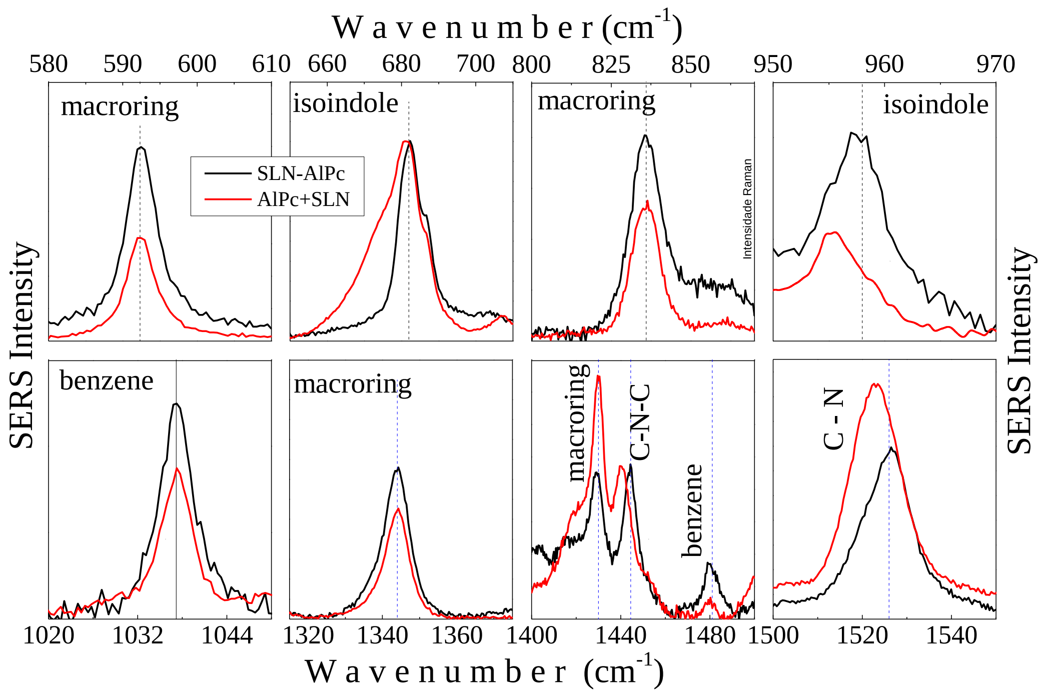

Figure S3. Zoom, at different spectral ranges, of the SERS spectra of SLN-AlPc formulation (black lines) and physical mixture (AlPc+SLN), (rede lines) of samples (i) Gly-solution and (ii) 2Gly@PEG/NPs, and (iv) normal Raman spectrum of Gly-solution.

(see Figure S3 - SLN-AlPc: black lines and AlPc+SL: red lines).

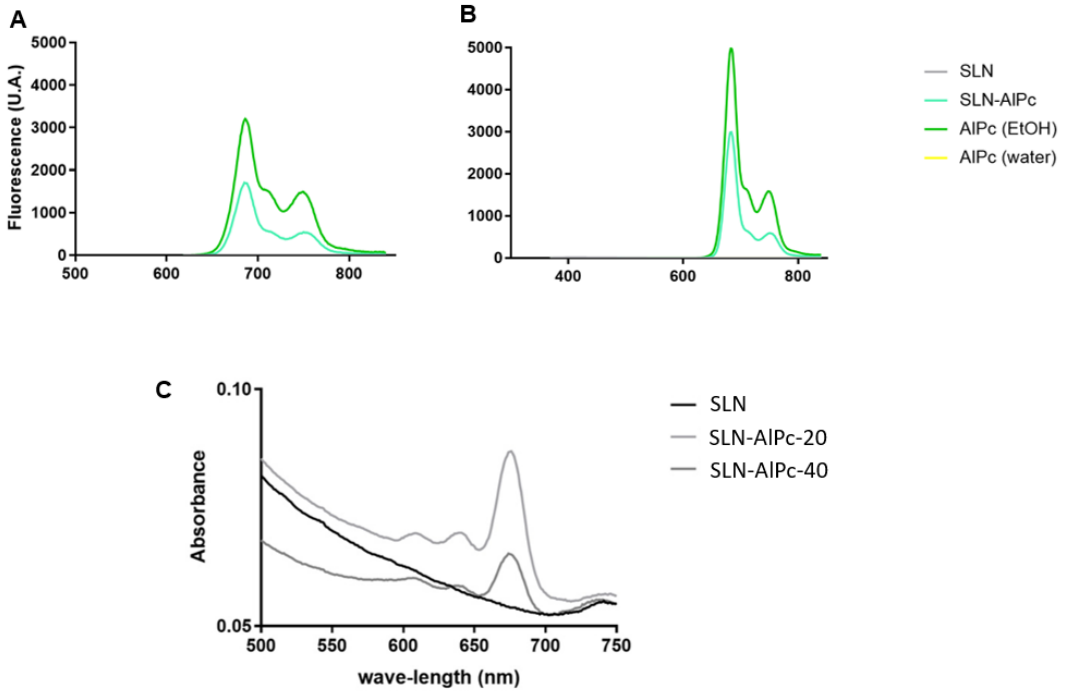

Figure S4. Fluorescence (**A, B**) and absorbance (**C**) spectrum of SLN-AlPc. A = Ex:660 nm and B = Ex:350 nm
